# A Comparative Analysis of Phenolic Content, Antioxidant Activity, Antimicrobial Activity, and Chemical Profile of *Coffea robusta* Extracts Using Subcritical Fluid Extraction and Supercritical Carbon Dioxide Extraction

**DOI:** 10.3390/foods12183443

**Published:** 2023-09-15

**Authors:** Pattarin Supanivatin, Aluck Thipayarat, Suwit Siriwattanayotin, Paweena Ekkaphan, Anat Deepatana, Jakrapop Wongwiwat

**Affiliations:** 1 Department of Food Engineering, King Mongkut’s University of Technology Thonburi, Bangkok 10140, Thailand; pattarin.s@mail.kmutt.ac.th (P.S.); athipaya@gmail.com (A.T.); suwit.sir@kmutt.ac.th (S.S.); 2 Scientific and Technological Research Equipment Centre, Chulalongkorn University, Bangkok 10330, Thailand; paweena.e@chula.ac.th; 3 Department of Chemical Engineering, Faculty of Engineering, Burapha University, Chonburi 20131, Thailand; anat@eng.buu.ac.th; 4 Department of Mechanical Engineering, King Mongkut’s University of Technology Thonburi, Bangkok 10140, Thailand

**Keywords:** HFC-134a, HCFC-22, phytonic extraction, supercritical extraction, total phenolic, antioxidant, antimicrobial

## Abstract

In this study, extracts of *Robusta*-roasted coffee were obtained using various extraction techniques, including subcritical fluid extractions using HFC-134a and HCFC-22 under room-temperature batch extraction, frozen-temperature batch extraction, and continuous extraction conditions. Additionally, supercritical carbon dioxide (SCCO_2_) extraction was performed using ethanol and tetrahydrofuran as co-solvents. These extractions were performed due to the presence of potent antioxidants and antibacterial substances in the extracts. Extraction machines were built to process the extraction. The antioxidant potential of the extracts was evaluated using total phenolic content and DPPH and FRAP assays, while antibacterial potential was identified using the disk diffusion method. The results showed that HCFC-22 extraction produced the highest yield compared to other extraction methods, but HFC-134a extraction had the highest antioxidant potential values. The yield and antioxidant potential of the extracts obtained using room-temperature batch extraction were slightly higher than those obtained using frozen-temperature batch extraction and continuous extraction. The yield and antioxidant potential of the extracts obtained using SCCO_2_ extraction were similar to those obtained using HFC-134a and HCFC-22 extractions, and co-solvents slightly improved the extraction performance. The extracts were found to be more effective as inhibitors of Gram-positive bacteria than Gram-negative bacteria. Caffeine was the most prominent tentative chemical compound in all coffee extracts. This research study provides a better understanding of various extraction techniques using HFC-134a, HCFC-22, and SCCO_2_ when applied to roasted *Robusta* coffee beans, with a focus on yield, antioxidant potential, antimicrobial potential, and tentative chemical profiles.

## 1. Introduction

Several natural antimicrobial agents have been identified that can extend the shelf life of fruits and food products. These agents include sulfur dioxide (SO_2_) in combination with chitosan [1], chlorine dioxide (ClO_2_) [2], and 1-methylcyclopropene (1-MCP) in combination with chlorine dioxide (ClO_2_) [3]. To address concerns about residual toxicity, natural compounds are being increasingly used instead of pure chemicals. Recently, essential oils extracted from herbs and plants have gained increasing interest as antibacterial and antioxidant agents [4,5,6,7], and several studies have explored their antimicrobial capabilities [8]. Thus, gaining a deeper understanding of the antibacterial properties of essential oils, including extracts from coffee, as well as their antioxidant activities, is crucial. The knowledge gained would offer a broad range of information for exploring different natural preservatives.

Worldwide, coffee is a highly consumed product that is known for its unique taste and aroma. In addition to their flavor profile, coffee bean extracts have been shown to possess antimicrobial and antioxidant properties, making them promising ingredients for cosmetic products [9,10]. Moreover, studies have suggested that regular coffee consumption is associated with lower blood pressure [11] and reduced abdominal obesity [12]. The roasting of coffee beans has been found to naturally enhance their antioxidant properties [13], and the degree of roasting can significantly affect the antioxidant potential of coffee extracts [14]. Although conventional extraction methods can be used to obtain coffee bean extracts, the process involves hydrolysis, thermolysis, and volatile loss, and the remaining solvents, such as hexane and dichloromethane, can be toxic [15]. Many organic solvents are coming under increased regulatory pressure.

The prototype of a phytonic extractor that utilizes tetrafluoroethane (HFC-134a or R134a) and hydrochlorofluorocarbon (HCFC-22 or R22) has been developed for extracting roasted coffee beans at an ambient temperature (below 40 degrees Celsius). This method uses an inert, non-flammable, non-corrosive, nontoxic, and highly selective solvent, along with a readily available and widely acceptable refrigerant, that is much simpler to use than organic solvents. Supercritical carbon dioxide extraction (SCCO_2_) is another well-known method for extracting coffee beans, and it is considered a moderately low-temperature extraction option by many researchers [16]. Some models of the SCCO_2_ approach have been studied [17,18], but the process requires at least 73 bar and up to 300 bar in some circumstances. In contrast, subcritical extraction requires lower temperatures and pressures.

Several compounds, such as procyanidins [19], β-carotenes [20], astaxanthin [21], and curcuminoids [22], have been extracted using HFC-134a. Although it is challenging to determine which extraction technique is better for roasted coffee, there is research evidence suggesting that HFC-134a, with its higher dipole moment than CO_2_ [23], may be more suitable for extracting polar molecules. In contrast, SCCO_2_ appears to be more appropriate for extracting non-polar molecules [24].

Therefore, we explored several extraction methods in this study, including subcritical HFC-134a extraction, subcritical HCFC-22 extraction, and SCCO_2_ extraction, to determine the optimal method for extracting roasted coffee with exquisite qualities. Robusta coffee is one of the most sought-after coffee types globally, and this study aims to determine which extraction technique yields the highest-quality coffee extract. To achieve this, the yield, antioxidant potential, antimicrobial potential, and chemical profiles of the obtained extracts were examined and compared using GC-MS.

## 2. Materials and Methods

### 2.1. Solvents

Both HFC-134a and HCFC-22 were purchased from Dot Bamboo (Bangkok) Co., Ltd., Bangkok, Thailand for subcritical extraction, and carbon dioxide (CO_2_) with 99.9% mass purity was purchased from Thai Special Gas Co., Ltd., Chonburi, Thailand for SCCO_2_ extraction.

### 2.2. Raw Materials

**Figure 1:**
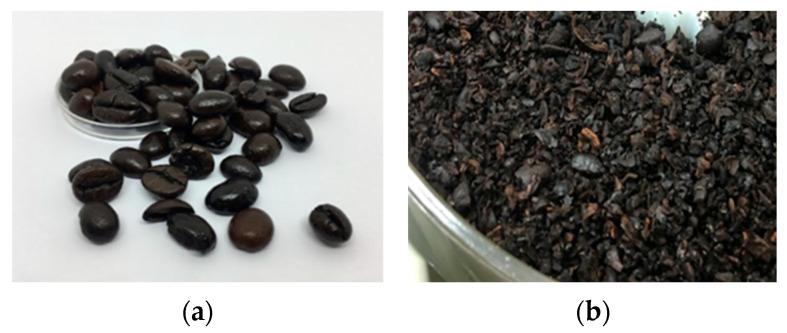
Roasted *Robusta* coffee beans: (**a**) coffee beans before grinding and (**b**) coffee beans after grinding.

To maintain the quality of coffee beans throughout the study, only commercially roasted coffee beans (*Robusta* coffee) from Chao Thai Pu Kao Co., Ltd., Chiang Mai, Thailand, were used. To obtain coffee beans with a diameter ranging from 1.0 to 1.5 mm, the coffee beans were ground in a bench coffee grinder (Tomex, TCG-108, Shanghai, China) and filtered as depicted in Figure 1a,b.

### 2.3. Extraction Process

#### 2.3.1. Subcritical Fluid Extraction Using HFC-134a and HCFC-22

**Figure 2:**
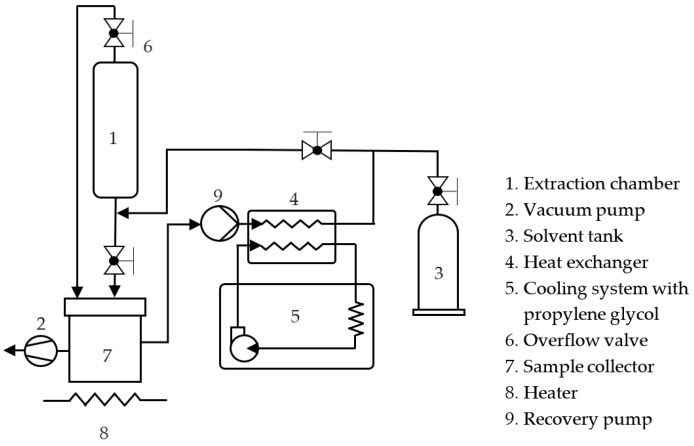
Schematic diagram of the apparatus for HFC-134a extraction and HCFC-22 extraction.

To conduct our roasted coffee extraction experiments, a custom-built subcritical extraction apparatus was utilized, as illustrated in Figure 2. HFC-134a and HCFC-22 were selected as the solvents. For the first step, an extraction chamber was used to hold the ground coffee samples, which were vacuumed to eliminate any remaining air. The solvent was then fed from the bottom into the extraction chamber through an overflow valve. Before being introduced to the extraction chamber, the solvent was cooled down in a heat exchanger using 20 °C propylene glycol from a separate liquid circulation loop. Batch extractions were conducted under two temperature treatments: room temperature (RT) at 25 °C and freezing (FR) conditions at −20 °C, with an extraction time of 60 min in both cases. For the continuous extraction experiment (Cont), a pure solvent was continuously circulated into the extraction chamber over the entire 60 min extraction period. Following the extraction step, the mixture of solvent and extract was dropped into the sample collector, where the solvent was completely separated before samples were taken. On average, it took less than 30 min for the solvent to evaporate. When the recovered solvent was ready for the next cycle of extraction, it was taken back into the solvent tank. Heat was applied to the bottom of the collector to increase the evaporation rate.

#### 2.3.2. SCCO_2_ Extraction

**Figure 3:**
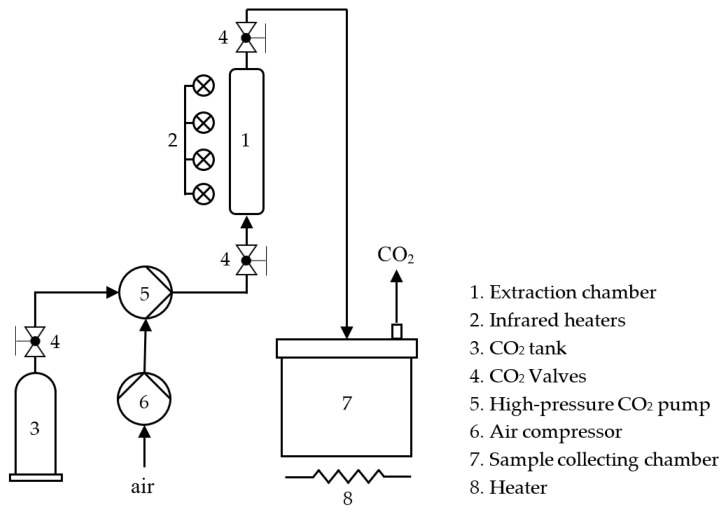
Schematic diagram of the SCCO_2_ extraction system.

The concept behind the supercritical fluid extraction system was similar to that of subcritical HFC-134a and HCFC-22 extraction in that the solvent was contained within an extraction chamber. However, this process was carried out at a much higher temperature and operating pressure. Our custom-built SCCO_2_ extraction apparatus, which is depicted in Figure 3, was used for the roast coffee extraction experiments. The extraction chamber’s open end was filled with ground coffee samples. Infrared heaters automatically maintain a constant temperature of 70 °C in the chamber. Liquid CO_2_ was then released from a tank and pumped using a pneumatic high-pressure CO_2_ pump into the extraction chamber. SCCO_2_ was produced by the high-pressure pump at 20 MPa, which was well past the CO_2_ critical point (7.38 MPa and 31.1 °C). The extraction process lasted 90 min before the coffee extracts were collected. Both the extracts and SCCO_2_ were moved from the extraction chamber to a sample collection chamber to obtain the coffee extracts. When the internal pressure in the collecting chamber was released, SCCO_2_ solidified into dry ice, which then instantaneously sublimated to CO_2_ gas aided by an external heat source. The bottom of the collecting chamber was used to gather the extracts. Before analyzing the total phenolic content, antioxidant and antimicrobial properties, and chemical profiles, the extracts were stored at −20 °C. Tetrahydrofuran (THF) and ethanol (EtOH) were used as co-solvents for the co-solvent treatments, and the co-solvent mixture was prepared before extraction in a plastic bag. A sample mixture consisting of 14 mL of co-solvent (either EtOH or THF) and 110 g of ground coffee was uniformly mixed before being added to the extraction chamber.

#### 2.3.3. Overall Extraction Yield

The overall extraction yield was calculated by calculating the ratio between the total extracted mass ($m_{e}$) and the initial mass of the raw material before extraction ($m_{i}$) using the following equation:

(1) $$extraction\;yield=\frac{m_{e}}{m_{i}}\times 100\%$$

### 2.4. Determination of Total Phenolic Contents

The Folin–Ciocalteu procedure was used to determine the total phenolic contents of the roasted coffee extracts [25,26,27]. A total of 500 µL of distilled water was mixed with 125 µL of the extract solution at a concentration of 10 mg/mL. Then, 125 µL of the Folin–Ciocalteu reagent (Loba Chemie Pvt. Ltd., Mumbai, India) was added to the solution and held for 6 min. Afterward, 1 mL of distilled water and 1.25 mL of sodium carbonate (7%) (Loba Chemie Pvt. Ltd., Mumbai, India) were added to the mixture. The reaction mixture was incubated at room temperature for 90 min in the dark before light absorption was measured at a wavelength of 765 nm using a microplate reader (M965 Metertech, Taipei, Taiwan). Gallic acid (Acros Organics, Morris Plains, NJ, USA) was used to create a calibration curve as a standard reference (concentration range: 0 to 0.5 mg/mL). The results were expressed as mg of gallic acid equivalent per g of extract (mg GAE/g extract).

### 2.5. Determination of Antioxidant Activity

#### 2.5.1. DPPH Assay

The 1,1-diphenyl-2-picrylhydrazy (DPPH) assay’s scavenging activity was evaluated using the steps outlined in the previous study [28]. A total of 100 μL of 0.2 mM DPPH solution (Sigma-Aldrich, Darmstadt, Germany) dissolved in methanol (QRëC, Auckland, New Zealand) was mixed with 50 μL of the sample solution. The reaction was carried out in a 96-well microplate (SPL Lifesciences, Co., Ltd., Gyeonggi-Do, Republic of Korea) and incubated at room temperature for 30 min in the dark. The microplate reader, used to measure absorbance, was set to 517 nm. As positive controls, gallic acid and ascorbic acid (Loba Chemie Pvt. Ltd., Mumbai, India) were utilized. The amount of extract required to inhibit DPPH by 50%, or the IC_50_ value (mg/mL), was used to express the scavenging activity.

#### 2.5.2. FRAP Assay 

The FRAP assay was measured using the methods proposed in previous studies [29,30] with some modifications. This method relies on reducing ferrous Fe^2+^ that forms at low pH by acetic acid (QRëC, New Zealand) from the complex of Fe^3+^ (FeCl_3_·6H_2_O) (QRëC, New Zealand) and TPTZ (2,4,6-tripyridyl-s-triazine) (Sigma-Aldrich, Buchs, Switzerland). Following this reduction, the change in absorbance at 595 nm is measured. The measurements were repeated three times. In the FRAP assay, the antioxidant potential of the samples was estimated from the FRAP data using a standard curve that was plotted using the FeSO_4_·7H_2_O (QRëC, New Zealand) linear regression equation. The sample readings were presented in µmol FeSO_4_/mg extract.

### 2.6. Antibacterial Activity

#### 2.6.1. Inoculum Preparation

The microbial testing for this study included 15 bacteria species: 7 Gram-positive species (*Bacillus cereus* (ATCC 11778), *Corynebacterium diphtheriae*, *Enterococcus faecalis* (ATCC 29212), *Enterococcus faecium* (UCLA 192), *Listeria innocua*, *Listeria monocytogenes,* and *Staphylococcus aureus* (ATCC 25923)), and 8 Gram-negative species (*Edwardsiella tarda*, *Escherichia coli* (ATCC 25922), *Klebsiella pneumoniae* (ATCC 27736), *Proteus mirabilis*, *Pseudomonas aeruginosa* (ATCC 27853), *Salmonella Typhimurium* (ATCC 13311), *Serratia marcescens,* and *Yersinia enterocolitica* (ATCC 27729)). All bacterial species were obtained from the Novel Food Processing Technology Laboratory, Department of Food Engineering, King Mongkut’s University of Technology, Thonburi, Bangkok.

Inoculums were prepared by transferring colonies of bacterial growth on Mueller–Hinton agar (MHA, Difco, Detroit, MI, USA) into individual tubes containing 0.85% normal saline solution. The density of the organism suspension was adjusted to a 0.5 McFarland turbidity standard (measured by McFarland Densitometer, Biosan, DEN-1B, Latvia) by adding more saline solution or more bacteria, which approximately corresponded to 1 × 10^8^ to 2 × 10^8^ CFU/mL.

#### 2.6.2. Determination of Inhibition Zone Using Disk Diffusion Assay

The antimicrobial activities of the extracts were first screened for their inhibitory zones using the agar disk diffusion method [31]. A sterile cotton swab was dipped into the suspension. Then, the inoculum was spread evenly over the entire MHA agar surface. A total of 20 µL of the extracts, which worked as antimicrobial agents, was dropped on filter paper discs with a diameter of 6 mm, and the filter paper discs were placed on the MHA plates. Discs impregnated with normal saline solution and DMSO (Loba Chemie Pvt. Ltd., Mumbai, India) were used as a negative control. Tetracycline (Oxoid, Basingstoke, UK) and chloramphenical (Oxoid, UK) were applied as control antibiotics for comparison. The plates were incubated at 35 ± 1 °C for 18 ± 2 h in an incubator (Benchmark, H2200-H, Suzhou, China). The inhibition zone’s diameter was measured using Vernier calipers (Total Tools, Suzhou, China), including the 6 mm diameter filter paper discs (Whatman, Hangzhou, China). The effectiveness of the extracts on antimicrobial activity increased with a larger inhibition zone of bacteria. Each antibacterial test was conducted with three replicates. The extracts with the highest solubility from different solvents in DMSO were selected for the determination of the inhibition zone.

### 2.7. Analysis of Chemical Profiles Using Gas Chromatography-Mass Spectrometry (GC-MS)

The extracts obtained via SCCO_2_ and subcritical extractions were evaluated in terms of their chemical profiles using the GC-MS method. The extracts were weighed and diluted to 20 mg/mL in dichloromethane (Merck, Darmstadt, Germany), and then the chromatographic analysis was performed using a 7890B GC system and a 7000C mass spectrometer (Agilent Technologies, Palo Alto, CA, USA) equipped with a HP-5ms capillary column (30 m × 0.25 mm × 0.25 µm film thickness, Agilent Technologies) to observe a broad range of chemical compounds from the extracts. A total of 1 µL of each extract sample diluted with dichloromethane was injected using an autosampler with a 20:1 split ratio. Helium was used as a carrier gas at a constant flow rate of 1.0 mL/min. The oven temperature program was initially maintained at 60 °C for 2 min before being ramped up to 5 °C/min up to 300 °C and finally held for 10 min at 300 °C. The temperature of the injector port and the GC-MS interface was set at 250 °C and 310 °C, respectively. The mass spectrometer was operated in the electron ionization (EI) mode with an electron energy of 70 eV, an ion source temperature of 250 °C, and a mass scan range of 33–550 *m*/*z*.

The identification of chemical compounds was performed by matching their mass spectra with the reference spectra in the NIST2011 library using MassHunter Qualitative Analysis Software, version B.06.01 SP1 (Agilent Technologies). Data processing was further carried out using Microsoft Excel 2020. In addition, for structural confirmation of some available compounds, the linear retention indices (LRI) were compared to those in the NIST2011 mass spectral library and the related literature [32]. The LRI value was calculated from the retention time of each peak in the sample in relation to the retention time of n-alkanes (C7-C37) under the same conditions according to the literature [33].

The quantification of each identified chemical component in terms of the relative peak area (%) was performed via area normalization measurement of the total ion chromatogram (TIC).

### 2.8. Data Analysis

The data are reported as (means ± standard error). The data were analyzed using a one-way analysis of variance and post hoc Duncan’s multiple range test. Statistical significance was considered at a *p*-value of <0.05 [34]. All statistical analyses were performed using SPSS^®^ software (version 17.0, SPSS Inc., Chicago, IL, USA).

## 3. Results

### 3.1. Extraction Yields

**Figure 4:**
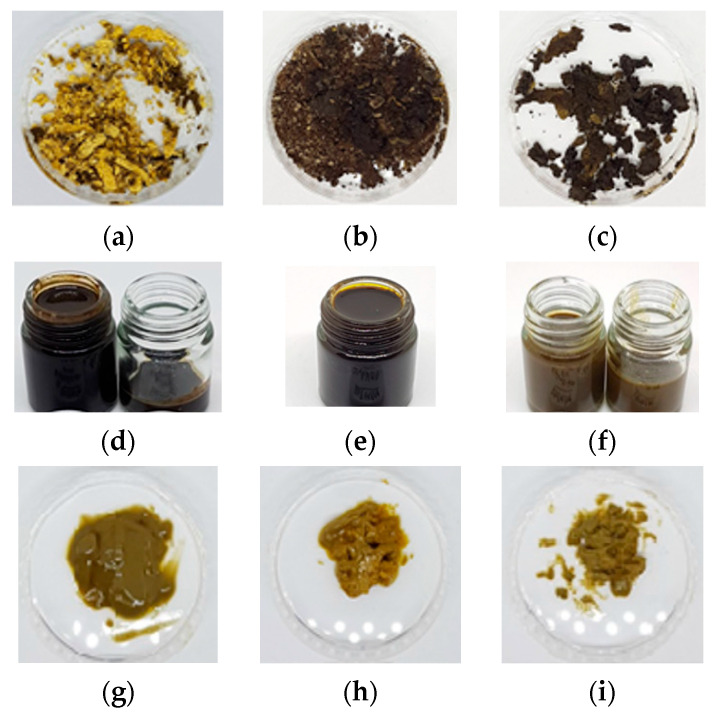
Extracts obtained using different extraction methods: (**a**) HFC-134a room-temperature batch extraction; (**b**) HFC-134a frozen-temperature batch extraction; (**c**) HFC-134a continuous extraction; (**d**) HCFC-22 room-temperature batch extraction; (**e**) HCFC-22 frozen-temperature batch extraction; (**f**) HCFC-22 continuous extraction; (**g**) SCCO_2_ without co-solvent; (**h**) SCCO_2_ with ethanol; (**i**) SCCO_2_ with tetrahydrofuran.

**Figure 5:**
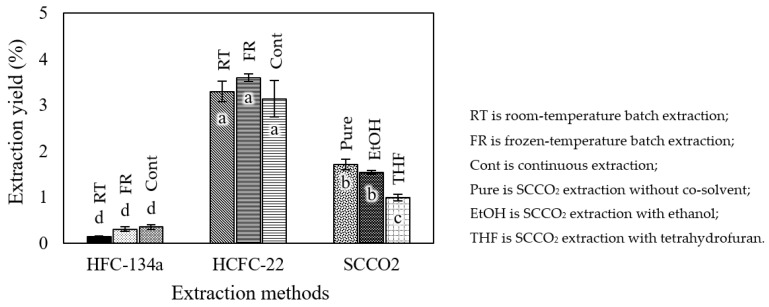
Roasted Robusta coffee extraction yields obtained using different extraction methods. Different letters on top of the bars imply that the values are significantly different (*p* < 0.05).

To determine the yield of the extracts of *Robusta*-roasted coffee beans, the total mass of the extracts was divided by the mass of the initial coffee beans before extraction. Figure 4 illustrates the extracts obtained using different extraction methods. The texture of the extracts obtained using HFC-134a appeared like small frozen powders; however, upon squeezing, the powder broke into oil. In contrast, the extracts obtained using HCFC-22, which provided the highest yield, were a dark brown liquid, while the extracts obtained using SCCO_2_ had a paste-like texture. Figure 5 shows the extraction yields obtained using various methods. Subcritical HCFC-22 extraction produced the highest yield, while HFC-134a extraction produced the lowest yield for roasted *Robusta* coffee beans. This indicates that HCFC-22 is more effective at extracting substances from coffee beans than other methods. Interestingly, the yields of extraction using HFC-134a and HCFC-22 did not show a significant difference when these extraction methods were used for room-temperature batch extraction, frozen-temperature batch extraction, and continuous extraction. The extraction yields obtained using HFC-134a ranged from 0.15 to 0.36 percent, while the yields obtained using HCFC-22 ranged from 3.14 to 3.60 percent. This suggests that HCFC-22 is more effective at extracting substances from roasted coffee beans. Pure SCCO_2_ extraction and SCCO_2_ extraction with EtOH did not show a significant difference in extraction yield, but they showed a significant difference compared to SCCO_2_ extraction with THF. Pure SCCO_2_ performed slightly better than SCCO_2_ with co-solvents. The extraction yields obtained using SCCO_2_ ranged from 0.99 to 1.71 percent.

### 3.2. Total Phenolic Content and Antioxidant Activity

**Figure 6:**
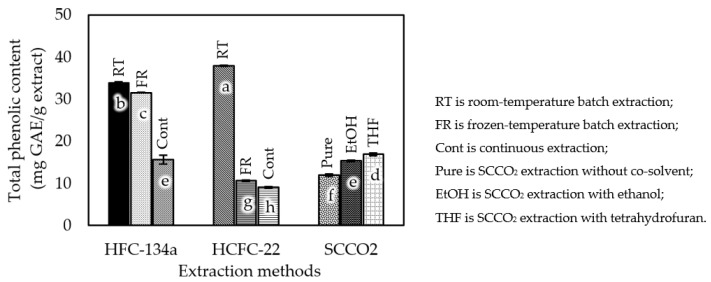
Total phenolic content of roasted *Robusta* coffee extracts obtained using different extraction methods. Different letters on top of the bars imply that the values are significantly different (*p* < 0.05).

The total phenolic content was determined by comparing the value with standard gallic acid. The results were expressed in terms of mg GAE/g extract. The results for each extraction method are presented in Figure 6. The total phenolic values ranged from 15.63 ± 1.09 to 33.87 ± 0.17 mg GAE/g extract for subcritical HFC-134a extraction; 9.05 ± 0.21 to 37.94 ± 0.14 mg GAE/g extract for HCFC-22 extraction; and 11.98 ± 0.21 to 16.90 ± 0.32 mg GAE/g extract for SCCO_2_ extraction. Subcritical extraction using HCFC-22 at room temperature provided the highest total phenolic content, at 37.94 ± 0.14 mg GAE/g extract. Extraction using HCFC-22 at room temperature showed a higher amount of total phenolic content than that obtained under the −20 °C condition. It was observed that the extraction temperature significantly affected the quantity of total phenolic content to some degree. The extraction temperature had a weaker effect on total phenolic content in the HFC-134a extraction process than in the HCFC-22 extraction process. However, the results also showed that batch extraction at room temperature yielded better total phenolic contents compared to continuous extraction in both cases. SCCO_2_ extractions with the co-solvents were able to improve the total phenolic contents of the extracts. Both the THF and EtOH experiments returned significantly higher total phenolic contents at 16.90 ± 0.32 and 15.35 ± 0.21 mg GAE/g extract (*p* < 0.05), respectively.

**Figure 7:**
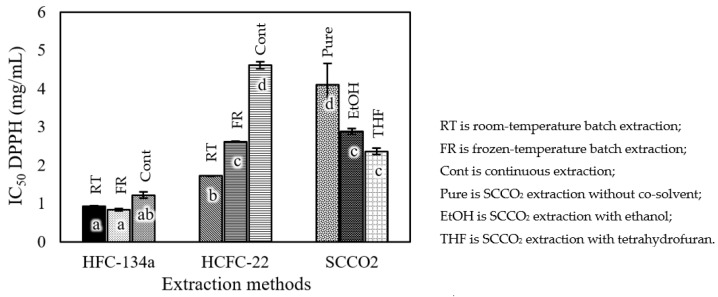
IC_50_ DPPH values of roasted *Robusta* coffee extracts obtained using different extraction methods. Different letters on top of the bars imply that the values are significantly different (*p* < 0.05).

One of the methods used to investigate antioxidant potential is the DPPH radical scavenging assay. This method is considered simple and sensitive, providing a better correlation between the concentration of the samples used and the percentage inhibition of DPPH. Figure 7 presents the IC_50_ values of gallic and ascorbic acids, which are 4.23 ± 0.07 and 15.17 ± 0.04 µg/mL, respectively, and were used as the positive controls for this experiment. In general, the IC_50_ values of the roasted coffee extracts obtained using subcritical HFC-134a extraction were lower than the values obtained using HCFC-22 extraction. The IC_50_ values of the extracts obtained using HFC-134a under room-temperature batch extraction, frozen-temperature batch extraction, and continuous extraction were not significantly different, ranging from 0.85 ± 0.03 to 1.22 ± 0.88 mg/mL. The IC_50_ of the extracts obtained using HCFC-22 under the room-temperature condition was lower than that obtained under the frozen condition and continuous condition, with values of 1.72 ± 0.01 mg/mL, 2.62 ± 0.02 mg/mL, and 4.61 ± 0.09 mg/mL, respectively. SCCO_2_ extraction with a co-solvent yielded significantly lower IC_50_ values compared to pure SCCO_2_ extraction. The IC_50_ values of the SCCO_2_ extracts ranged from 2.36 ± 0.08 to 4.11 ± 0.56 mg/mL, indicating that SCCO_2_ extraction with THF provided a lower IC_50_ value than SCCO_2_ extraction with EtOH.

**Figure 8:**
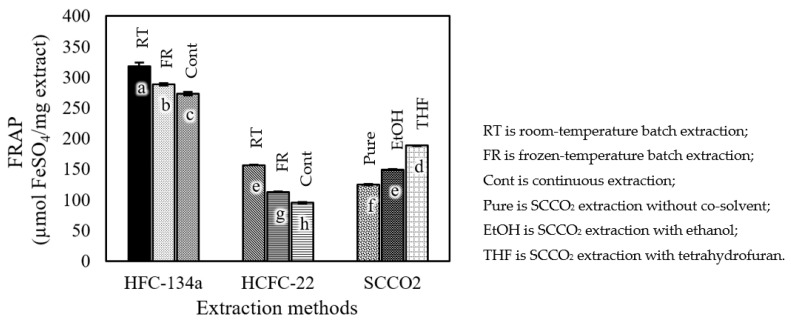
FRAP values of roasted coffee extracts obtained using different extraction methods. Different letters on top of the bars imply that the values are significantly different (*p* < 0.05).

The FRAP assay is another well-known method to identify the antioxidant power of extracts. The higher the absorbance, the greater the concentration of antioxidant compounds in a sample. The FRAP assay results are shown in Figure 8. The extraction process with HFC-134a provided the highest FRAP values from 273.38 ± 2.83 to 317.58 ± 6.26 µmol FeSO_4_/mg extract, while the extraction process with HCFC-22 provided lower FRAP values from 95.16 ± 1.22 to 156.66 ± 0.66 µmol FeSO_4_/mg extract than HFC-134a. For both methods, the extraction process at room temperature provided better FRAP values compared to the frozen or continuous conditions. The FRAP values of extracts obtained under frozen-temperature batch extraction were lower than the FRAP values of extracts obtained under room-temperature batch extraction for both HFC-134a and HCFC-22 extractions. Lastly, the SCCO_2_ extraction process with a co-solvent could significantly enhance the FRAP values, with either THF or EtOH as the co-solvent.

### 3.3. Antibacterial Activity of Roasted Coffee Extracts

To assess the antibacterial properties of the roasted coffee extracts, a disk diffusion method was utilized with 6 mm diameter disks to analyze the negative controls, antibiotic controls, and samples. As indicated in Table 1, the roasted coffee extracts exhibited potent antibacterial activity against *B. cereus*, *L. innocua,* and *E. tarda*. Nevertheless, *K. pneumoniae*, *S. Typhimurium,* and *P. aeruginosa* proved to be more resistant strains against the roasted coffee extracts. When clear zones are observed and compared, it is found that the relationship between the extracts of *Robusta* coffee beans and types of bacteria is still unclear. However, it appears that the coffee bean extracts from all extraction methods possess greater antibacterial efficacy against Gram-positive bacteria than Gram-negative bacteria.

### 3.4. Tentative Chemical Profiles

**Figure 9:**
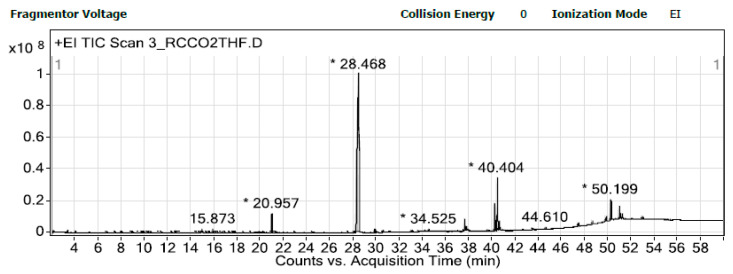
GC-MS chromatogram of coffee extract obtained via SCCO_2_ extraction with THF. * Numbers on peaks represent retention time of volatile compounds.

The gas chromatography-mass spectrometry (GC-MS) method was employed to identify the compounds present in the coffee bean extracts. Figure 9 provides an example of the tentative peaks of the chemical compounds detected via the GC-MS analysis, while Table 2 presents the tentative compounds obtained from three different extraction methods, including HFC-134a extraction at room temperature, HCFC-22 extraction at room temperature, and SCCO_2_ extraction with THF. These three extraction methods were selected based on their ability to produce extracts with the best antioxidant properties. Caffeine, a well-known compound found in all coffee types, was the major tentative compound obtained in all the extraction methods, with the HFC-134a extraction producing the highest percentage of caffeine (73.43%), followed by SCCO_2_ extraction (68.51%) and HCFC-22 extraction (22.87%). The other compounds detected in the extracts were found in significantly smaller amounts compared to caffeine, such as 5-pregnen-3β-ol-20-one, propionate, 5-pregnen-3β-ol-20-one, butyrate that were found between 2 and 4 percent, 5-pregnen-3β-ol-20-one, propionate, 5-pregnen-3β-ol-20-one, and butyrate that were found between 7 and 8 percent.

In addition to the listing of individual compounds, the chemical compounds detected were grouped into 18 major categories, which are presented at the end of Table 2. One of the major groups identified in all extraction methods was purines, which include caffeine. Terpenes and terpenoids were the second most prominent compounds in the extracts, with similar amounts detected using different extraction methods, comprising approximately 13% of the total compounds. Furthermore, small quantities of phenolic compounds with antioxidant potential were also observed in the extracts. To demonstrate the agreement between the compounds in the coffee extracts and the database, the retention index (RI) of each compound was calculated using the retention time of the compound and the alkane standard retention time. The RI values are provided in the table. Several compounds in the coffee extracts exhibited antioxidant properties due to the presence of aromatic rings. Notably, some unknown compounds that were not presented in the GC-MS database were mostly detected in the extracts obtained using HCFC-22 extraction when compared to the extracts obtained using HFC-134a extraction and SCCO_2_ extraction.

## 4. Discussion

### 4.1. Comparison of Extraction Yields

The various extraction methods employed in this study resulted in different extraction yields. The highest extraction yield was obtained using the HCFC-22 extraction, followed by the SCCO_2_ extraction and the HCF-134a extraction, respectively. The yield of coffee bean extracts obtained using the SCCO_2_ extraction was around 2%, which was consistent with a previous study where the extraction temperature was around 65 °C [35]. However, the yield could be greater than 10% at higher extraction pressures [36,37]. To the best of our knowledge, the subcritical HFC-134a extraction and the subcritical HCFC-22 extraction are not common for coffee extraction, unlike the SCCO_2_ extraction. However, there was a comparison of HFC-134a and SCCO_2_ for palm mesocarp oil extraction that showed the oil yield obtained using the SCCO_2_ extraction was higher than the HFC-134a extraction due to the better mass transfer characteristics around the solvent critical point [38].

Some studies explained the yield of subcritical fluid extraction from the standpoint of dipole moment values [39,40,41]. Table 3 summarizes the dipole moment values of the three solvents used in this study, with HFC-134a having the highest value, followed by HCFC-22 and CO_2_. Normally, solvents with higher dipole moments are more soluble in water. However, subcritical fluid extraction using HCFC-22 produced the highest overall extraction yield from roasted coffee beans, which was even better than SCCO_2_ extraction. This suggests that the solubility of the oil-like extracts in HCFC-22 was greater than that in HFC-134a in subcritical fluid extraction and SCCO_2_ extraction. Hence, the chemical compositions of the extracts obtained using different techniques presumably should be distinguishable and were later compared using chromatography, antioxidant, total phenolic, and antimicrobial techniques. However, regarding the final yields of the extracts obtained using the same technique, the extraction modes and temperatures were slightly different among the different treatments.

### 4.2. Total Phenolic Content and Antioxidant Activity

The highest extraction yield was obtained using the subcritical fluid extraction with HCFC-22, whereas the extracts obtained using HFC-134a exhibited the highest antioxidant potential. The IC_50_ values obtained from the DPPH assay were comparable to previous research on *Robusta* coffee [14]. Based on the values of dipole moments, it is most likely that antioxidant substances in roasted coffee beans could be dissolved by solvents with a high dipole moment, such as HFC-134a. Room-temperature batch extraction in subcritical fluid extraction showed slightly better performance compared to frozen-temperature batch extraction for both HFC-134a and HCFC-22. Both solvents stayed in the mixture phase in the extraction chamber, but the saturated pressures of the solvents at room temperature were higher than under freezing conditions, at approximately 4 bars for HFC-134a and 7 bars for HCFC-22. These pressures could enhance the extraction of coffee beans, which has been mentioned in some previous studies examining the effects of varying extraction pressures [21,41]. Despite SCCO_2_ having the lowest dipole moment [39], the addition of EtOH and THF enhanced the extraction of antioxidant compounds from roasted coffee beans, with THF yielding higher-quality extracts than EtOH. All results regarding total phenolic content, IC_50_ values from the DPPH assay, and FRAP values also seemed to be related to each other in the evaluation of antioxidant potential.

### 4.3. Antimicrobial Activity

In a previous study, it was found that essential oils can cause a leakage of ions and cell contents from bacteria [8] and are highly effective against Gram-positive bacteria over Gram-negative bacteria, similar to another experimental study on inhibition zones [43]. It was also reported that Gram-negative bacteria are covered by a lipopolysaccharide layer that helps protect these bacteria from antibacterial substances [44]. In this study, the *Robusta* roasted coffee extracts could perform as antibacterial substances against Gram-positive bacteria better than Gram-negative bacteria.

### 4.4. Chemical Profiles

The GC-MS results revealed that the majority of the chemical compounds were caffeine, which belongs to the purine group and is commonly found in coffee extracts [45]. However, the amount of caffeine detected in this study was considerably higher than in some previous studies [46,47,48]. A previous study also reported that *Robusta* seeds contain 40–50% more caffeine than *Arabica* seeds [49]. The tentative compound analysis in this study showed a significant amount of 5-pregnen-3β-ol-20-one, propionate, and 5-pregnen-3β-ol-20-one, butyrate contents, which were not commonly found in other studies and are considered to be antioxidant substances. Furthermore, although terpenes and terpenoids were the second most commonly found compounds in this study, they were not mentioned in previous works, where the major compounds were reported to be from the pyrazine and furan groups [50,51]. Additionally, our research also found that certain phenolic compounds, including phenol, catechol, and 4-ethylcatechol, were thermally degraded under different roasting conditions by chlorogenic acid, an essential compound present in green beans [52].

## 5. Conclusions

The extraction of Robusta coffee was explored using three different extraction methods: subcritical HFC-134a, subcritical HCFC-22, and supercritical carbon dioxide (SCCO_2_) extractions. An extraction machine was built for subcritical extraction, and another extraction machine was built for supercritical extraction. For subcritical extraction, the conditions varied between room-temperature batch extraction, frozen-temperature batch extraction, and continuous flow extraction. For supercritical extraction, CO_2_ was chosen as the main solvent, and either ethanol (EtOH) or tetrahydrofuran (THF) was selected as the co-solvent. From the results, it was found that subcritical HCFC-22 extraction provided the highest overall extraction yield. Room-temperature batch extraction provided a slightly higher yield than frozen-temperature batch extraction and continuous extraction. Room-temperature batch extraction is a preferred condition because it requires less energy for extraction than other conditions. The SCCO_2_ extraction yield was between the yields obtained using HCFC-22 extraction and HFC-134a extraction.

The antioxidant properties of the extracts obtained using different extraction methods were also examined via total phenolic content, DPPH, and FRAP assays. The concentration of the extracts was varied from 0 to 10 mg/mL to measure the values of total phenolic content and the percent inhibition of DPPH. Overall, subcritical HFC-134a extraction seemed to perform better in terms of antioxidant properties, except for HCFC-22 room-temperature batch extraction, which had the highest total phenolic content. The overall yield and antioxidant properties of the extracts obtained using SCCO_2_ extraction were somewhere between those obtained using subcritical HFC-134a and subcritical HCFC-22 extraction, while CO_2_ with THF performed better than CO_2_ with EtOH and CO_2_ without a co-solvent.

Antimicrobial activities were also examined in this study by testing the inhibition zone of bacteria on a disk. The results showed that Gram-positive bacteria were more affected by the *Robusta* coffee extracts than Gram-negative bacteria. Different extraction methods did not seem to have significant differences in antimicrobial performance, which might require some further study.

Gas chromatography-mass spectrometry (GC-MS) was used to identify the compounds present in the roasted *Robusta* coffee extracts obtained using three different extraction methods: HFC-134a at room temperature, HCFC-22 at room temperature, and SCCO_2_ with THF. Caffeine was the major compound found in all extracts obtained using the three different extraction methods, while terpenes and terpenoids were the second most prominent compounds found in the coffee extracts.

## Figures and Tables

**Table 1:** The antibacterial activities of the roasted coffee extracts obtained using different extraction methods, against selected microorganisms in terms of inhibition zones (mm) based on the disk diffusion method.

Microbial Strain	Antibiotic Control		HFC-134a (25% Coffee Extracts)			HCFC-22 (50% Coffee Extracts)			SCCO_2_ (33% Coffee Extracts)		
	Chloram- phenicol	Tetra- cycline	RT	FR	Cont	RT	FR	Cont	SCCO_2_	SCCO_2_ + EtOH	SCCO_2_ + THF
**Gram-positive**											
*B. cereus*	27.0	33.0	8.0	7.0	8.0	6.0	7.0	8.0	10.0	9.0	9.0
*C. diphtheriae*	38.0	35.0	20.0	15.0	16.0	12.0	10.0	NI	16.0	18.0	19.0
*E. faecalis*	24.0	20.0	NI	NI	NI	NI	NI	8.0	8.0	8.0	8.0
*E. faecium*	29.0	30.0	NI	7.0	NI	7.0	7.0	NI	10.0	9.0	9.0
*L. innocua*	33.0	40.0	10.0	9.3	8.0	7.0	8.7	7.3	10.0	9.0	9.0
*L. monocytogenes*	33.0	39.0	NI	NI	NI	NI	NI	NI	8.0	8.0	8.0
*S. aureus*	26.0	33.0	8.0	8.0	8.0	6.0	8.0	NI	8.0	8.0	8.0
**Gram-negative**											
*E. tarda*	21.0	15.0	8.0	7.0	7.0	7.0	7.0	9.0	10.0	8.0	8.0
*E. coli*	21.3	11.0	6.0	NI	NI	NI	NI	NI	NI	NI	NI
*K. pneumonia*	25.0	20.0	NI	NI	NI	NI	NI	NI	NI	NI	NI
*S. typhimurium*	26.0	26.0	NI	NI	NI	NI	NI	NI	NI	NI	NI
*S. marcescens*	22.0	13.0	NI	NI	NI	NI	NI	NI	8.0	NI	NI
*P. mirabillis*	20.0	NI	NI	8.0	7.0	NI	NI	11.0	NI	NI	NI
*P. aeruginosa*	18.0	17.0	NI	NI	NI	NI	NI	NI	NI	NI	NI
*Y. enterocolitica*	22.0	24.0	8.0	7.0	8.0	NI	7.0	7.0	11.0	10.0	10.0

The negative control was 0.85% NSS and 100% DMSO. NI—no inhibition zone. RT—room-temperature batch extraction; FR—frozen-temperature batch extraction; Cont—continuous flow extraction; EtOH—ethanol; THF—tetrahydrofuran.

**Table 2:** Tentative chemical compounds of *Robusta* coffee obtained via HFC-134a extraction at room temperature, HCFC-22 extraction at room temperature, and SCCO_2_ extraction with THF.

Name	RT (Min)	LRI		CAS No.	Content (%)		
		Exp ^a^	Ref ^b^		HFC-134a	HCFC-22	SCCO_2_ w/THF
2-Hexanol ^2^	3.35	809	803	108-11-2	0.10	0.31	0.19
Butanoic acid, 3-methyl- ^1^	3.65	826	827	503-74-2	0.02	-	-
2-Furanmethanol ^6^	4.04	849	845	98-00-0	0.09	0.10	0.16
Phenol ^11^	6.74	978	978	108-95-2	0.07	0.02	0.08
2-Furanmethanol, acetate ^6^	7.14	995	998	623-17-6	0.01	0.02	-
Pyrazine, 2-ethyl-6-methyl- ^13^	7.23	999	997	13925-03-6	0.01	0.02	-
Pyrazine, trimethyl- ^13^	7.34	1003	1008	14667-55-1	0.01	-	-
1H-Pyrrole-2-carboxaldehyde ^16^	7.44	1007	1009	1003-29-8	0.01	-	0.03
2-Cyclopenten-1-one, 2-hydroxy-3-methyl- ^9^	7.94	1025	1022	80-71-7	0.03	-	-
D-Limonene ^17^	8.06	1029	1029	5989-27-5	-	-	0.03
Ethanone, 1-(1H-pyrrol-2-yl)- ^16^	8.85	1059	1060	1072-83-9	0.10	-	0.11
1H-Pyrrole, 3-ethyl-2,4-dimethyl- ^16^	9.27	1074	1075	517-22-6	0.02	-	-
Pyrazine, 3-ethyl-2,5-dimethyl- ^13^	9.41	1079	1078	13360-65-1	0.04	0.02	-
Pyrazine, 2-ethyl-3,5-dimethyl- ^13^	9.62	1087	1085	13925-07-0	0.02	-	-
Phenol, 2-methoxy- ^11^	9.69	1089	1092	90-05-1	0.20	0.05	0.08
3-Pyridinol ^14^	9.93	1098	NR	109-00-2	-	-	0.12
Pyrazine, (1-methylethenyl)- ^13^	10.17	1107	1092	38713-41-6	0.04	-	
Maltol ^15^	10.28	1111	1110	118-71-8	0.10	-	0.13
2-Cyclopenten-1-one, 3-ethyl-2-hydroxy- ^9^	10.48	1118	1121	21835-01-8	0.09	-	0.08
Nicotinyl alcohol ^14^	10.57	1121	1119	100-55-0	0.03	-	0.03
Methyl nicotinate ^14^	11.06	1139	1140	93-60-7	0.02	-	-
5H-5-Methyl-6,7-dihydrocyclopentapyrazine ^13^	11.11	1140	1147	23747-48-0	0.02	-	-
3-Pyridinol, 6-methyl- ^14^	11.44	1152	NR	1121-78-4	-	-	0.04
2,4,6-Trimethyl-1,3-phenylenediamine ^3^	11.60	1158	1159	3102-70-3	0.04	-	-
1H-Pyrrole, 1-(2-furanylmethyl)- ^16^	12.32	1184	1185	1438-94-4	0.11	-	-
Pyrazine, 2-methyl-3-(2-propenyl)- ^13^	12.61	1194	NR	55138-62-0	0.06	-	-
Catechol ^11^	12.67	1196	1200	120-80-9	-	-	0.19
Ethanone, 1-(2,5-dihydroxyphenyl)- ^11^	14.84	1276	NR	490-78-8	0.15	-	0.10
Phenol, 4-ethyl-2-methoxy- ^11^	14.95	1280	1287	2785-89-9	0.90	0.15	0.35
Indole ^8^	15.32	1293	1295	120-72-9	0.09	0.02	0.05
Furan, 2,2’-[oxybis(methylene)]bis- ^6^	15.57	1303	1305	4437-22-3	0.11	0.01	-
Ethanone, 1-(2-hydroxy-5-methylphenyl)- ^11^	15.88	1314	1316	1450-72-2	1.20	0.14	0.42
5-Aminoindole ^8^	16.26	1329	NR	5192-03-0	0.05	-	0.02
2-Naphthalenol, 5-amino- ^18^	16.73	1347	NR	86-97-5	0.04	-	0.02
4-Hydroxy-7-methyl-1,8-naphthylidine ^10^	16.91	1354	NR	1569-18-2	0.10	-	-
4-Ethylcatechol ^11^	17.64	1382	1392	1124-39-6	-	0.01	0.11
2-Cyclohexen-1-one, 4-hydroxy-3,5,5-trimethyl-4-(3-oxo-1-butenyl)- ^17^	17.80	1388	NR	7070-24-8	0.17	-	0.07
p-tert-Butyl catechol ^11^	20.26	1487	1493	98-29-3	0.06	-	-
Butylated Hydroxytoluene ^11^	20.96	1516	1514	128-37-0	-	0.04	2.01
5-tert-Butylpyrogallol ^11^	21.25	1528	1526	20481-17-8	0.05	-	-
4-(2,4-Dimethoxyphenyl)butan-2-one ^11^	23.73	1635	NR	93467-61-9	0.04	-	-
Benzenemethanol, 3-hydroxy-5-methoxy- ^11^	27.55	1812	NR	30891-29-3	0.08	-	-
2-Propenoic acid, 3-(4-hydroxy-3-methoxyphenyl)-, methyl ester ^11^	28.13	1840	1854	2309-07-1	0.11	-	0.03
Caffeine ^12^	28.63	1865	1848	58-08-2	73.43	22.87	68.51
Hexadecanoic acid, methyl ester ^4^	29.86	1927	1926	112-39-0	1.25	0.15	0.38
Pyrrolo [1,2-a]pyrazine-1,4-dione, hexahydro-3-(2-methylpropyl)- ^16^	30.07	1938	1908	5654-86-4	0.38	0.02	0.14
n-Hexadecanoic acid ^1^	30.57	1964	1963	57-10-3	0.20	-	0.29
Hexadecanoic acid, ethyl ester ^4^	31.17	1995	1993	628-97-7	0.12	0.01	0.08
9,12-Octadecadienoic acid (Z,Z)-, methyl ester ^4^	33.04	2095	2092	112-63-0	0.62	0.05	0.18
Oleic acid ^1^	33.14	2101	2102	112-80-1	0.27	-	0.07
Methyl stearate ^4^	33.61	2128	2128	112-61-8	0.18	0.03	0.07
Linoleic acid ethyl ester ^4^	34.24	2163	2163	544-35-4	0.17	-	0.07
Hexadecanamide ^10^	34.53	2179	2182	629-54-9	0.11	0.10	0.27
Eicosanoic acid, methyl ester ^4^	37.07	2329	2335	1120-28-1	0.18	-	-
9-Octadecenamide, (Z)- ^10^	37.76	2371	2375	301-02-0	0.33	0.59	0.77
5-Pregnen-3.beta.-ol-20-one, propionate ^17^	40.18	2525	2487	1000368-37-1	2.85	4.34	3.53
5-Pregnen-3.beta-ol-20-one, butyrate ^17^	40.42	2541	2546	1000368-37-2	8.14	7.73	8.07
Squalene ^17^	44.61	2832	2847	111-02-4	0.25	-	-
Stigmasterol ^17^	50.20	3268	3249	83-48-7	-	-	3.00
Acids ^1^					0.49	-	0.36
Alcohols ^2^					0.10	0.31	0.19
Anilines ^3^					0.04	-	-
Esters ^4^					2.52	0.24	0.78
Furanones ^5^					-	-	-
Furans ^6^					0.21	0.13	0.16
Hydrocarbons ^7^					-	-	-
Indoles ^8^					0.14	0.02	0.07
Ketones ^9^					0.12	-	0.08
Miscellaneous nitrogen compounds ^10^					0.54	0.69	1.04
Phenolic compounds ^11^					2.86	0.41	3.37
Purines ^12^					73.43	22.87	68.51
Pyrazines ^13^					0.20	0.04	-
Pyridines ^14^					0.05	-	0.19
Pyrones ^15^					0.10	-	0.13
Pyrroles ^16^					0.62	0.02	0.28
Terpenes and terpenoids ^17^					11.41	12.07	14.70
Others ^18^					0.04	-	0.02

NR—not report; Exp ^a^—experimental linear retention indices calculated using n-alkane standards on a HP-5 column; Ref ^b^—linear retention indices obtained from the National Institute of Standards and Technology (NIST) database (webbook.nist.gov/chemistry, accessed on 17 May 2023). Superscript numbers indicate 18 major categories of tentative chemical compounds.

**Table 3:** Dipole moment of HFC-1347a, HCFC-22, and CO_2_ [23,39,42].

Solvent	Formula	Dipole Moment (D)
HFC-134a	CH_2_FCF_3_	2.67
HCFC-22	CHF_2_Cl	1.40
CO_2_	CO_2_	0.00

## Data Availability

The data used to support the findings of this study can be made available by the corresponding author upon request.

## References

[1] Zhang J., Hao X., Li X., Wang W. (2017). Effect of chitosan coating combined with sulfur dioxide fumigation on the storage quality of fresh areca nut. J. Food Process. Preserv..

[2] Zhang X.-M., Fu M.-R. (2018). Inhibitory effect of chlorine dioxide (ClO_2_) fumigation on growth and patulin production and its mechanism in Penicillum expansum. LWT.

[3] Yang X., Zhang X., Fu M., Chen Q., Muzammil J.M. (2018). Chlorine dioxide fumigation generated by a solid releasing agent enhanced the efficiency of 1-MCP treatment on the storage quality of strawberry. J. Food Sci. Technol..

[4] Bevilacqua A., Speranza B., Perricone M., Sinigaglia M., Corbo M.R. (2017). Bioactivity of essential oils towards fungi and bacteria: Mode of action and mathematical tools. Essential Oils in Food Processing: Chemistry, Safety and Applications.

[5] Bosquez-Molina E., Ronquillo-de Jesús E., Bautista-Baños S., Verde-Calvo J., Morales-López J. (2010). Inhibitory effect of essential oils against *Colletotrichum gloeosporioides* and *Rhizopus stolonifer* in stored papaya fruit and their possible application in coatings. Postharvest Biol. Technol..

[6] Mehdi M., Asgar A., Alderson P.G. (2010). Effect of cinnamon oil on incidence of anthracnose disease and postharvest quality of bananas during storage. Int. J. Agric. Biol..

[7] Singh P., Pandey A.K., Sonker N., Tripathi N. (2011). Preservation of Buchnania lanzan Spreng. seeds by *Ocimum canum* Sims. essential oil. Ann. Plant Prot..

[8] Burt S. (2004). Essential oils: Their antibacterial properties and potential applications in foods—A review. Int. J. Food Microbiol..

[9] Raba D.N., Poiana M.-A., Borozan A.B., Stef M., Radu F., Popa M.-V. (2015). Investigation on crude and high-temperature heated coffee oil by ATR-FTIR spectroscopy along with antioxidant and antimicrobial properties. PLoS ONE.

[10] Bessada S.M., Alves R.C., Oliveira M.B.P.P. (2018). Coffee silverskin: A review on potential cosmetic applications. Cosmetics.

[11] Han B., Nazary-Vannani A., Talaei S., Clark C.C., Rahmani J., Rasekhmagham R., Kord-Varkaneh H. (2019). The effect of green coffee extract supplementation on blood pressure: A systematic review and meta-analysis of randomized controlled trials. Phytother. Res..

[12] Roshan H., Nikpayam O., Sedaghat M., Sohrab G. (2018). Effects of green coffee extract supplementation on anthropometric indices, glycaemic control, blood pressure, lipid profile, insulin resistance and appetite in patients with the metabolic syndrome: A randomised clinical trial. Br. J. Nutr..

[13] Acidri R., Sawai Y., Sugimoto Y., Handa T., Sasagawa D., Masunaga T., Yamamoto S., Nishihara E. (2020). Phytochemical profile and antioxidant capacity of coffee plant organs compared to green and roasted coffee beans. Antioxidants.

[14] Trinh N.T.N., Tuan N.N., Thang T.D., Kuo P.-C., Thanh N.B., Tam L.N., Tuoi L.H., Nguyen T.H., Vu D.C., Ho T.L. (2022). Chemical Composition Analysis and Antioxidant Activity of Coffea robusta Monofloral Honeys from Vietnam. Foods.

[15] Yuwono S., Hanasasmita N., Sunarharum W. (2019). Effect of different aroma extraction methods combined with GC-MS on the aroma profiles of coffee. IOP Conference Series: Earth and Environmental Science.

[16] Essien S.O., Young B., Baroutian S. (2020). Technology, Recent advances in subcritical water and supercritical carbon dioxide extraction of bioactive compounds from plant materials. Trends Food Sci..

[17] Cornelio-Santiago H.P., Gonçalves C.B., de Oliveira N.A., de Oliveira A.L. (2017). Supercritical CO_2_ extraction of oil from green coffee beans: Solubility, triacylglycerol composition, thermophysical properties and thermodynamic modelling. J. Supercrit. Fluids.

[18] Getachew A.T., Saravana P.S., Cho Y.J., Woo H.C., Chun B.S. (2018). Concurrent extraction of oil from roasted coffee (*Coffea arabica*) and fucoxanthin from brown seaweed (*Saccharina japonica*) using supercritical carbon dioxide. J. CO2 Util..

[19] Tan L., Zhu L., Feng Z. (2018). Technical Study on Extraction of Procyanidins in *Lycium ruthenicum* Murr. Using Sub-critical Fluid 1, 1, 1, 2-Tetrafluoroethane (R134a) and Content Determination from Different Producing Areas. J. Med. Plant.

[20] Mustapa A., Manan Z., Azizi C.M., Setianto W., Omar A.M. (2011). Extraction of β-carotenes from palm oil mesocarp using sub-critical R134a. Food Chem..

[21] Han Y., Ma Q., Wang L., Xue C. (2012). Extraction of astaxanthin from Euphausia pacific using subcritical 1,1,1,2-tetrafluoroethane. J. Ocean Univ. China.

[22] Kwon H.-L., Chung M.-S. (2015). Pilot-scale subcritical solvent extraction of curcuminoids from *Curcuma long* L.. Food Chem..

[23] Corr S. (2005). 1, 1, 1, 2-tetrafluoroethane (R-134a): A selective solvent for the generation of flavor and fragrance ingredients. Natural Flavors and Fragrances.

[24] Sang J., Wang H., Jin J., Meng H. (2017). Comparison and modelling of rutin solubility in supercritical carbon dioxide and subcritical 1, 1, 1, 2-tetrafluoroethane. J. CO2 Util..

[25] Srisook K., Salee P., Charoensuk Y., Srisook E. (2010). In vitro anti-oxidant and anti-tyrosinase activities of the rhizomal extractsfrom Amomum biflorum Jack. Thai J. Bot..

[26] Rodglin C., Srisook E., Srisook K. (2017). Effects of Extraction Conditions on Total Phenolic Content, Total Flavonoid Content and Antioxidant Activities of Different Parts of *Citrus aurantium* L.. Burapha Sci. J..

[27] Kantawong F., Singhatong S., Srilamay A., Boonyuen K., Mooti N., Wanachantararak P., Kuboki T. (2017). Properties of macerated herbal oil. BioImpacts BI.

[28] Klaokwan S., Doungnapa B., Rattiya B., Panadda S., Yaowaluck C., Ekaruth S. (2012). Antioxidant and anti-inflammatory activities of hot water extract from Pluchea indica Less. herbal tea. J. Med. Plant Res..

[29] Wanyo P., Meeso N., Siriamornpun S. (2014). Effects of different treatments on the antioxidant properties and phenolic compounds of rice bran and rice husk. Food Chem..

[30] Pokorná J., Venskutonis P., Kraujalyte V., Kraujalis P., Dvořák P., Tremlová B., Kopřiva V., Ošťádalová M. (2015). Comparison of different methods of antioxidant activity evaluation of green and roast C. Arabica and C. Robusta coffee beans. Acta Alimentaria.

[31] The European Society of Clinical Microbiology and Infectious Diseases (2019). Antimicrobial Susceptibility Testing EUCAST Disk Diffusion Method.

[32] Adams R.P. (2007). Identification of Essential Oil Components by Gas Chromatography/Mass Spectrometry.

[33] Van Den Dool H., Kratz P.D. (1963). A generalization of the retention index system including linear temperature programmed gas-liquid partition chromatography. J. Chromatogr. A.

[34] Duangjai A., Suphrom N., Wungrath J., Ontawong A., Nuengchamnong N., Yosboonruang A. (2016). Comparison of antioxidant, antimicrobial activities and chemical profiles of three coffee (*Coffea arabica* L.) pulp aqueous extracts. Integr. Med. Res..

[35] Hurtado-Benavides A., Dorado D., del Pilar Sánchez-Camargo A. (2016). Study of the fatty acid profile and the aroma composition of oil obtained from roasted Colombian coffee beans by supercritical fluid extraction. J. Supercrit. Fluids.

[36] Prokopchuk D., Kostenko M., Pokrovskiy O. (2021). Spontaneous Precipitation of Caffeine from Supercritical Extracts of Roasted Coffee Beans. Theor. Found. Chem. Eng..

[37] Muangrat R., Pongsirikul I. (2019). Recovery of spent coffee grounds oil using supercritical CO_2_: Extraction optimisation and physicochemical properties of oil. CYTA J. Food.

[38] Setapar S.H.M., Khatoon A., Ahmad A., Yunus M.-A.C., Zaini M.A.A. (2014). Use of supercritical CO_2_ and R134a as a solvent for extraction of β-carotene and α-tocopherols from crude palm oil. Asian J. Chem..

[39] Li S., Ong C., Lee M., Lee H. (1990). Supercritical fluid extraction and chromatography of steroids with Freon-22. J. Chromatogr..

[40] Lu J., Feng X., Han Y., Xue C. (2014). Optimization of subcritical fluid extraction of carotenoids and chlorophyll a from Laminaria japonica Aresch by response surface methodology. J. Sci. Food Agric..

[41] Gbashi S., Adebo O.A., Piater L., Madala N.E., Njobeh P.B. (2017). Subcritical water extraction of biological materials. Sep. Purif. Rev..

[42] Karamoddin M., Varaminian F. (2013). Solubility of R22, R23, R32, R134a, R152a, R125 and R744 refrigerants in water by using equations of state. Int. J. Refrig..

[43] Hasheminya S.-M., Dehghannya J. (2020). Composition, phenolic content, antioxidant and antimicrobial activity of Pistacia atlantica subsp. kurdica hulls’ essential oil. Food Biosci..

[44] Sihite N.W., Rusmarilin H., Rotua M. (2021). Analysis of The Phytochemical Characteristics of Jasmine Flower Against Escherichia coli and Staphylococcus aureus. Microbiol. Indones..

[45] Sulewska A.M., Larsen F.H., Sørensen J.K., Pedersen A.H. (2021). Advanced instrumental characterization of the coffee extracts produced by pilot scale instant coffee process. Eur. Food Res. Technol..

[46] Thammarat P., Kulsing C., Wongravee K., Leepipatpiboon N., Nhujak T. (2018). Identification of volatile compounds and selection of discriminant markers for elephant dung coffee using static headspace gas chromatography—Mass spectrometry and chemometrics. Molecules.

[47] Kalschne D.L., Viegas M.C., De Conti A.J., Corso M.P., de Toledo Benassi M. (2018). Steam pressure treatment of defective Coffea canephora beans improves the volatile profile and sensory acceptance of roasted coffee blends. Food Res. Int..

[48] Dippong T., Dan M., Kovacs M.H., Kovacs E.D., Levei E.A., Cadar O. (2022). Analysis of Volatile Compounds, Composition, and Thermal Behavior of Coffee Beans According to Variety and Roasting Intensity. Foods.

[49] Burdan F., Preedy V.R. (2015). Caffeine in coffee. Coffee in Health and Disease Prevention.

[50] Cordoba N., Fernandez-Alduenda M., Moreno F.L., Ruiz Y. (2020). Technology, Coffee extraction: A review of parameters and their influence on the physicochemical characteristics and flavour of coffee brews. Trends Food Sci. Technol..

[51] Dong W., Hu R., Long Y., Li H., Zhang Y., Zhu K., Chu Z. (2019). Comparative evaluation of the volatile profiles and taste properties of roasted coffee beans as affected by drying method and detected by electronic nose, electronic tongue, and HS-SPME-GC-MS. Food Chem..

[52] Shibamoto T., Preedy V.R. (2015). Volatile chemicals from thermal degradation of less volatile coffee components. Coffee in Health and Disease Prevention.

